# Feature genes identification and immune infiltration assessment in abdominal aortic aneurysm using WGCNA and machine learning algorithms

**DOI:** 10.3389/fcvm.2024.1497170

**Published:** 2024-11-12

**Authors:** Ming Xie, Xiandeng Li, Congwei Qi, Yufeng Zhang, Gang Li, Yong Xue, Guobao Chen

**Affiliations:** ^1^ Department of Pharmacy, Jiangyin Hospital of Traditional Chinese Medicine, Jiangyin Hospital Affiliated to Nanjing University of Chinese Medicine, Jiangyin, Jiangsu, China; ^2^ College of Pharmacy, Chongqing Medical University, Chongqing, China; ^3^ Department of Pharmacy, Jianhu County People’s Hospital, Jianhu, Jiangsu, China; ^4^ Department of Vascular Surgery, The Second Affiliated Hospital of Shandong First Medical University, Tai’an, Shandong, China; ^5^ Postdoctoral Workstation, Shandong First Medical University & Shandong Academy of Medical Sciences, Jinan, Shandong, China; ^6^ Department of Pulmonary and Critical Care Medicine, Jiangyin Hospital of Traditional Chinese Medicine, Jiangyin Hospital Affiliated to Nanjing University of Chinese Medicine, Jiangyin, Jiangsu, China; ^7^ Department of Cardiology, Jiangyin Hospital of Traditional Chinese Medicine, Jiangyin Hospital Affiliated to Nanjing University of Chinese Medicine, Jiangyin, Jiangsu, China

**Keywords:** abdominal aortic aneurysm, feature gene, machine learning, WGCNA, immune cell infiltration

## Abstract

**Objective:**

Abdominal aortic aneurysm (AAA) is a life-threatening vascular condition. This study aimed to discover new indicators for the early detection of AAA and explore the possible involvement of immune cell activity in its development.

**Methods:**

Sourced from the Gene Expression Omnibus, the AAA microarray datasets GSE47472 and GSE57691 were combined to generate the training set. Additionally, a separate dataset (GSE7084) was designated as the validation set. Enrichment analyses were carried out to explore the underlying biological mechanisms using Disease Ontology, Kyoto Encyclopedia of Genes and Genomes, and Gene Ontology. We then utilized weighted gene co-expression network analysis (WGCNA) along with 3 machine learning techniques: least absolute shrinkage and selection operator, support vector machine-recursive feature elimination, and random forest, to identify feature genes for AAA. Moreover, data were validated using the receiver operating characteristic (ROC) curve, with feature genes defined as those having an area under the curve above 85% and a *p*-value below 0.05. Finally, the single sample gene set enrichment analysis algorithm was applied to probe the immune landscape in AAA and its connection to the selected feature genes.

**Results:**

We discovered 72 differentially expressed genes (DEGs) when comparing healthy and AAA samples, including 36 upregulated and 36 downregulated genes. Functional enrichment analysis revealed that the DEGs associated with AAA are primarily involved in inflammatory regulation and immune response. By intersecting the result of 3 machine learning algorithms and WGCNA, 3 feature genes were identified, including MRAP2, PPP1R14A, and PLN genes. The diagnostic performance of all these genes was strong, as revealed by the ROC analysis. A significant increase in 15 immune cell types in AAA samples was observed, based on the analysis of immune cell infiltration. In addition, the 3 feature genes show a strong linkage with different types of immune cells.

**Conclusion:**

Three feature genes (MRAP2, PPP1R14A, and PLN) related to the development of AAA were identified. These genes are linked to immune cell activity and the inflammatory microenvironment, providing potential biomarkers for early detection and a basis for further research into AAA progression.

## 1 Introduction

Abdominal aortic aneurysm (AAA) is a vascular disease characterized by the abnormal dilation of the abdominal aorta. It is associated with the destruction and loss of elasticity of the arterial wall and primarily affects men over 40 years old, especially those with common risk elements such as smoking, raised blood pressure, and elevated cholesterol (1, 2). If AAA is not treated promptly, it increases the risk of aortic rupture, leading to severe bleeding and even posing a threat to the patient's life. Studies have revealed that AAA is a leading contributor to unexpected deaths among older adults (3). Therefore, early screening and diagnosis of AAA patients are crucial to prevent AAA rupture.

The development of AAA is multifaceted, encompassing the breakdown of elastin, changes in collagen structure and the involvement of inflammatory cells (4, 5). The inflammatory response is a critical factor in initiating and sustaining the progression of AAA, with the resulting series of pathological changes ultimately leading to aneurysm formation (6). There has been a growing appreciation for the contribution of immune and inflammatory factors to AAA development. Continuous inflammation leads to AAA formation and progression through the degradation and remodeling of the components of the vascular wall (7). The interplay between various immune cell types creates a complex inflammatory environment that promotes AAA development (8–10). The inflammatory microenvironment's role in AAA development also makes it a potential target for early detection. Identifying related immune responses and markers could improve screening and enable earlier intervention, reducing rupture risk (11). Considering the significant role of inflammation in AAA progression, integrating bioinformatics approaches provides a necessary and logical progression. These techniques, such as machine learning and co-expression network analysis, allow us to uncover the genetic and molecular mechanisms underlying the immune responses and inflammation associated with AAA.

Recent advancements in microarray-based integrated bioinformatics analyses have significantly enhanced the discovery of essential genes linked to specific diseases, providing promising candidates for diagnostic biomarkers (12). Weighted Gene Co-expression Network Analysis (WGCNA) is a systems biology technique that categorizes genes with similar functions into modules based on their expression relationships, thus revealing the complex organization of the genome (13). Unlike strategies that rely on differentially expressed genes (DEGs) analysis, its advantage lies in organizing genes into modules and connecting them to disease traits or biological processes, ultimately identifying key genes in disease pathways.

Publicly available gene expression profiles of AAA patients from 3 datasets were extracted from the Gene Expression Omnibus (GEO) database. Two of these datasets were fused to construct a training set, while the remaining dataset was used for validation. Then we utilized a variety of machine learning methods to identify AAA feature genes. A validation cohort confirmed gene validity, and the receiver operating characteristic (ROC) curve assessed prediction ability. Finally, through quantitative analysis, we explored the infiltration of various immune cell subsets within AAA patient tissues and delved into the correlations between these subsets and their associated gene expression profiles. This research sheds new light on the immunopathological mechanisms of AAA, offering crucial clues for subsequent research on targeted treatments.

## 2 Materials and methods

### 2.1 Data gathering and evaluation

The primary outcome was the classification of each sample as either “AAA” or “Control” group based on gene expression data. AAA was defined as an abdominal aortic diameter exceeding 3.0 cm, confirmed through abdominal ultrasound screening, with additional evaluation using CT or MRI in more complex cases or for pre-surgical planning (1). We explored the GEO database (14) for raw data related to AAA and eventually downloaded 3 datasets that examined AAA tissue samples from both patients and healthy participants: GSE47472 (controls: 8, AAA patients: 14) (Supplementary Table 1), GSE57691 (controls: 10, AAA patients: 49) (Supplementary Table 2), and GSE7084 (controls: 10, AAA patients: 9) (Supplementary Table 3). Table 1 presents the characteristics of the datasets.

**Table 1:** Overview of the datasets used in this study.

Database	Dataset	Platform	Sample
GEO	GSE57691	GPL10558	10 controls and 49 AAA cases
GEO	GSE47472	GPL10558	8 controls and 14 AAA cases
GEO	GSE7084	GPL570, GPL2507	10 controls and 9 AAA cases

We merged the gene expression data from GSE47472 and GSE57691 into a new matrix, which we designated as the training data (Supplementary Table 4). The datasets GSE47472 and GSE57691 were selected as the training set because of their larger sample sizes and shared platform, which ensured data consistency and supported robust machine learning analysis. GSE7084, although smaller and from a different platform, was used as an independent validation set to confirm the reliability and generalizability of our findings. These datasets were chosen for their relevance to AAA and the availability of both patient and control samples, allowing for comprehensive comparative analysis. Batch effects were addressed using the “sva” package (15), and datasets had samples excluded when inter-group discrepancies were not resolved (Supplementary Figure S1).

### 2.2 Differential gene analysis

The “limma” package (16) in R software was used to analyze variations in gene expression in AAA patients compared to control subjects, applying an adjusted (adj) *p*-value of <0.05 and |log2 fold change (FC)| > 1 to identify DEGs. Genes exhibiting a log2FC > 1 and an adj *p*-value <0.05 were categorized as up-regulated, reflecting increased expression, while genes with log2FC < −1 and the same *p*-value threshold were marked as down-regulated, indicating reduced expression. The pheatmap and volcano plot were used to display the selected DEGs.

### 2.3 Pathway enrichment evaluation

In this work, the R packages “clusterProfiler” and “DOSE” were used to perform functional enrichment analysis of DEGs, utilizing Gene Ontology (GO), Kyoto Encyclopedia of Genes and Genomes (KEGG) and Disease Ontology (DO) (17–19). Pathways related to the genes were explored using KEGG enrichment analysis. GO enrichment analysis was divided into 3 categories: molecular function (MF), cellular component (CC), and biological process (BP). Moreover, DO enrichment analysis was applied to study diseases associated with the genes. Using a q-value of less than 0.05 as a threshold, enrichment analysis was performed to explore biological functions, detect pathway enrichments, and assess disease associations.

### 2.4 Construction of WGCNA target module and feature genes screening

To uncover gene networks and co-expressed gene modules potentially relevant to the disease, we utilized WGCNA (20). This method was applied to identify gene modules linked to clinical traits. First, we calculated the variance of each gene and selected those with a standard deviation greater than 0.7 for further analysis. Clustering analysis was then performed on all samples, and outlier samples were removed based on clustering distance. A soft threshold (β = 9) was selected based on the network's topological properties to construct a scale-free co-expression network, transforming the expression matrix into an adjacency matrix and subsequently into a topological overlap matrix (TOM). To identify gene co-expression modules, we employed average linkage hierarchical clustering based on the TOM, with a hybrid dynamic tree-cutting algorithm determining the module boundaries. To ensure the robustness of the identified modules, a minimum size threshold of 60 genes was set. Subsequently, we calculated the eigengene for each module to capture its overall expression pattern. We then performed clustering analysis to merge modules with similar eigengenes, applying a merging threshold of 0.25. Key genes were identified using high gene significance (GS) and module membership (MM) scores, and gene module-clinical trait associations were visualized with the “ComplexHeatmap” package (21).

### 2.5 Machine learning based feature gene screening

We implemented 3 machine learning methods in this research, leveraging the R packages “glmnet”, “e1071”, and “randomForest”. First, we applied Least Absolute Shrinkage and Selection Operator (LASSO) logistic regression for feature selection, using L1 regularization to identify the most important features (22). Next, the Support Vector Machine—Recursive Feature Elimination (SVM-RFE) method, which recursively eliminates irrelevant features, was used to iteratively remove less significant features and determine the optimal variables (23). Finally, the Random Forest (RF) algorithm was used for classification, regression, and feature selection by building multiple decision trees, aggregating their results, and providing a robust evaluation of feature importance while handling noisy data (24). These methods were used to analyze the intersecting genes from these analyses, and feature genes were identified based on their importance in the intersecting set.

### 2.6 Feature gene validation

We systematically analyzed key gene expression profiles in AAA samples compared to normal controls using the R software to assess their diagnostic significance. Initially, we began by conducting expression variation analysis with the “limma” package, followed by generating box plots using the “ggpubr” package (25) to visually depict the differences in variation in core gene expression across groups. Furthermore, ROC curves for each core gene were constructed using the “pROC” package (26), and we calculated the area under the ROC curve (AUC) values with 95% confidence intervals to quantify diagnostic accuracy. A higher AUC value, approaching 1, indicates stronger diagnostic capability for AAA. Finally, to validate the robustness of these core genes, we used the external validation dataset GSE7084 and re-evaluated their expression patterns and diagnostic value through box plots and ROC curves across different datasets.

### 2.7 Immune cell infiltration analysis

The immune environment is key to understanding immune cell composition and function, providing insights for predicting disease progression and evaluating treatment efficacy. We utilized single-sample gene set enrichment analysis (ssGSEA) (27) to determine the expression patterns of 28 immune cell types in the samples under study. Differences in the abundance of each immune cell type between the AAA group and the control group were compared using the Wilcoxon rank-sum test, with *p* < 0.05 as the criterion for identifying immune cell types with higher infiltration levels. Moreover, Spearman correlation coefficients were used to analyze the relationship between immune cell abundance and gene expression levels in the samples, with *p* < 0.05 considered indicative of a significant regulatory relationship between immune cells and genes.

### 2.8 Statistical methods

R software version 4.2.2 was used for statistical analysis. For data meeting the criteria of normal distribution and equal variances, comparisons between the two groups were performed using a *t*-test or *U*-test. Correlations were assessed using Pearson's correlation or Spearman's correlation test, with statistical significance defined as a *p*-value less than 0.05.

## 3 Results

### 3.1 Identification of DEGs

**Figure 1:**
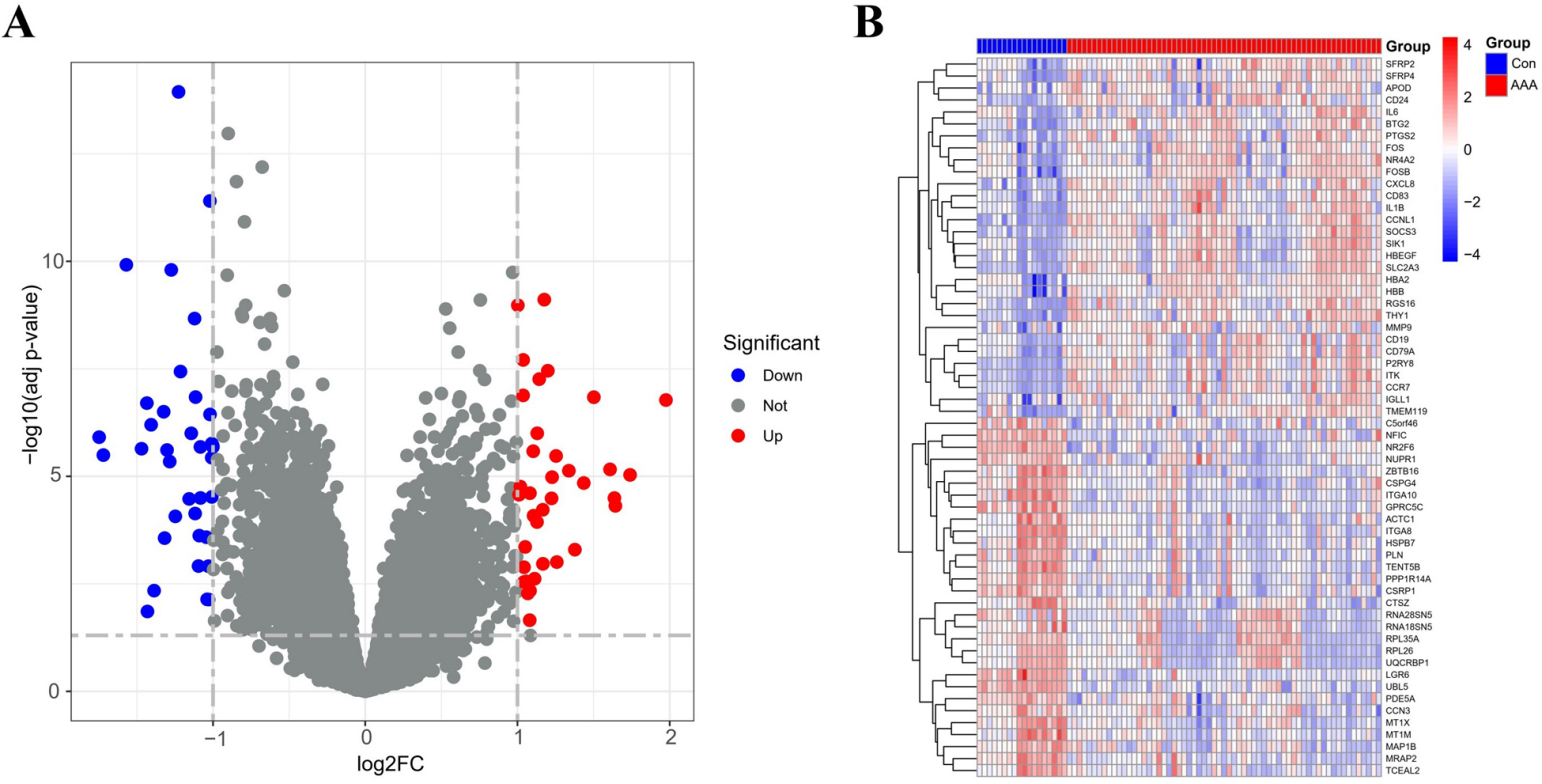
DEGs between AAA patients and controls. **(A)** Volcano plots display all DEGs in the training dataset, with blue spots indicating down-regulated genes and red spots signifying up-regulated genes. **(B)** The heatmap displays DEGs between control and AAA groups. Samples from AAA patients are highlighted in red, whereas those from normal controls are in blue. Genes with increased expression levels are marked by red blocks, and blue blocks signify genes with decreased expression levels.

By applying the “limma” package, differential gene expression analysis of the merged dataset identified 72 DEGs, based on the criteria of an adj p-value <0.05 and absolute |log2FC| > 1 (Supplementary Table 5). Of these, 36 genes were upregulated (log2FC > 1) and 36 were downregulated (log2FC < −1), as illustrated in the volcano plot (Figure 1A). The top 60 DEGs have been indicated in Figure 1B.

### 3.2 DO, GO functional analysis and KEGG pathway enrichment

**Figure 2:**
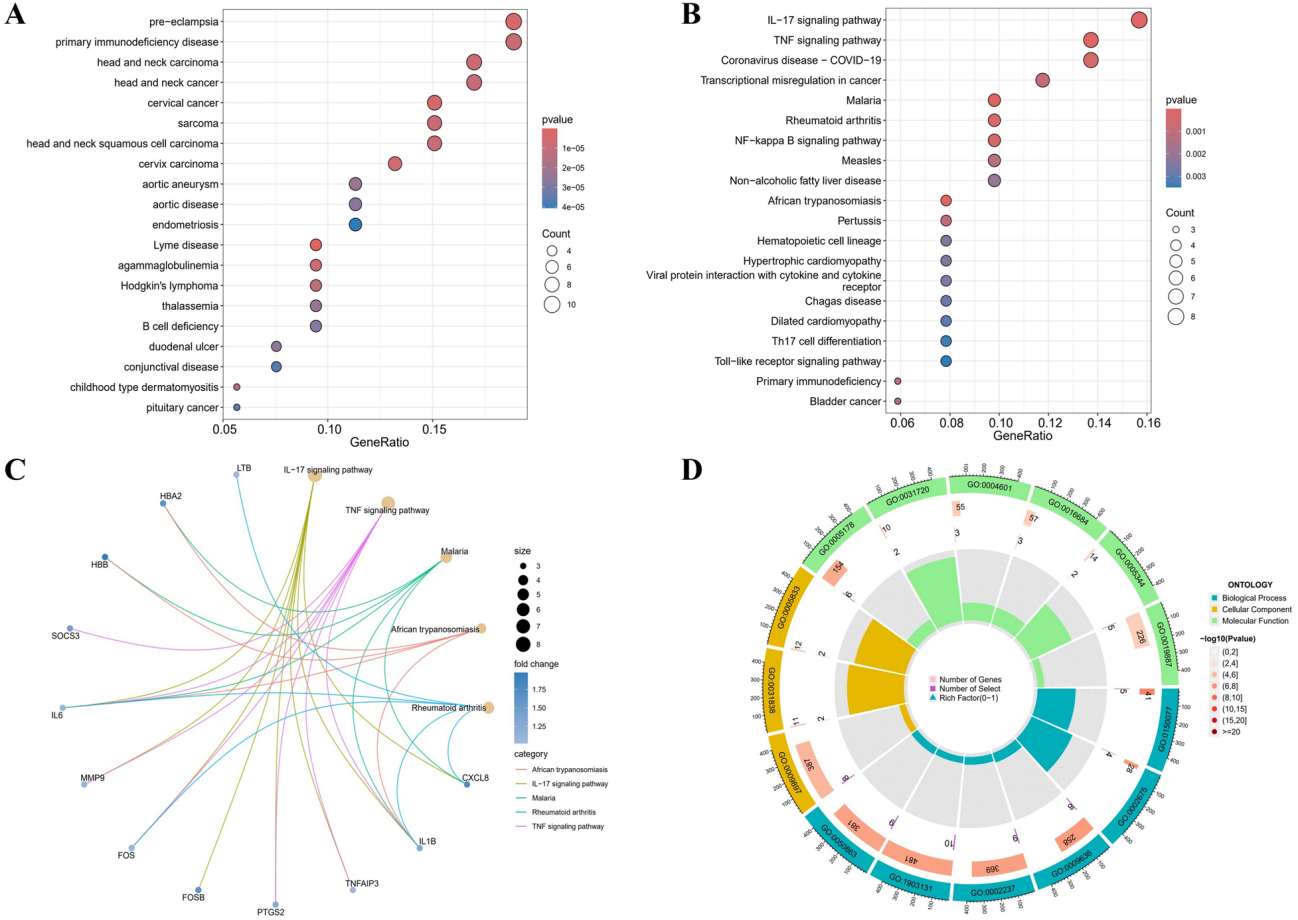
Functional enrichment profiling of DEGs. **(A)** The DO enrichment analysis is illustrated using a bubble diagram, highlighting the top 20 significantly enriched gene entries. **(B)** Bubble plots are used to display the KEGG enrichment analysis, featuring the top 20 pathways with the highest significance. **(C)** Results of KEGG are depicted on circle charts. **(D)** GO analysis of characteristic gene modules.

According to the DO analysis, these DEGs were involved in diseases like pre-eclampsia, primary immunodeficiency disease, head and neck carcinoma, cervical cancer and aortic aneurysm (Figure 2A) (Supplementary Table 6). KEGG pathway analysis highlighted a strong involvement of these DEGs in the interleukin 17 (IL-17) signaling pathway, the tumor necrosis factor (TNF) signaling pathway, the transcriptional misregulation in cancer, the rheumatoid arthritis and other pathways (Figures 2B,C) (Supplementary Table 7). Enrichment was considered significant with q-values <0.05. Finally, GO functional annotation revealed enrichment in 15 terms among the DEGs (Figure 2D).

Notable enrichment of DEGs was observed in the GO BP analysis for pathways including the regulation of neuroinflammatory response (GO: 0150077), the positive regulation of acute inflammatory response (GO: 0002675), and the response to toxic substance (GO: 0009636) among others. GO CC enrichment analysis identified a marked enrichment of DEGs in the external side of plasma membrane (GO: 0009897), the haptoglobin-hemoglobin complex (GO: 0031838) and the hemoglobin complex (GO: 0005833). The GO MF enrichment analysis revealed significant DEGs enrichment in the integrin binding (GO: 0005178), the haptoglobin binding (GO: 0031720), and the peroxidase activity (GO: 0004601) among others (Supplementary Table 8). All GO terms and pathways were considered statistically significant with q-values <0.05. These enriched pathways, especially IL-17 and TNF signaling, are key to inflammatory processes that degrade the aortic wall, driving AAA progression.

### 3.3 WCGNA analysis and identification of significant modules

**Figure 3:**
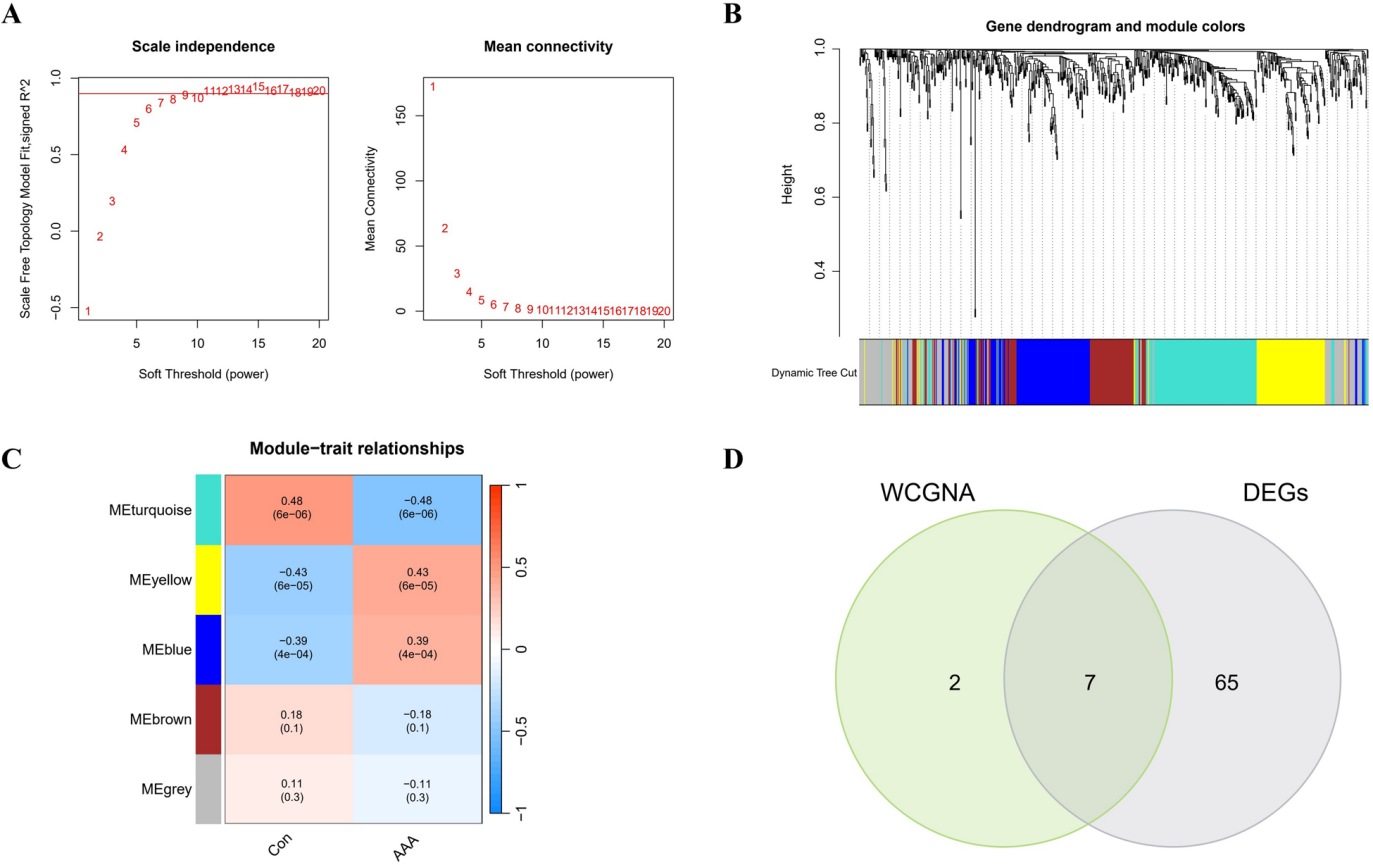
WGCNA analysis applied to AAA. **(A)** Gene correlations best match scale-free topology when β is set to 9 in soft-threshold analysis. **(B)** Using average linkage clustering, the gene dendrogram shows module assignments from dynamic tree cutting below. **(C)** Relationship analysis between identified modules and AAA. **(D)** Overlap of AAA DEGs and feature genes presented in a Venn diagram through WGCNA.

In this research, WGCNA was used to group genes closely linked to AAA, and all samples were incorporated into the analysis after screening (Supplementary Figure S2). To establish a scale-free network, we used the soft threshold method and determined the optimal threshold of 9 based on the R^2^ = 0.9 criterion (Figure 3A). Subsequently, we performed modular analysis on the resulting network and merged modules according to the cutoff value, ultimately identifying 5 biologically significant co-expression modules (Figure 3B). Furthermore, a significant positive correlation (cor) is observed between the MEturquoise module and AAA (cor=0.48, *p* = 6e-06) (Figure 3C). The final selection included 9 genes from the MEturquoise module, which had cor.MM values higher than 0.8 and cor.GS values above 0.5, marking them as targeted genes. We overlapped genes acquired from WGCNA and 72 DEGs and obtained 7 candidate genes for AAA (Figure 3D).

### 3.4 Selection of notable genes

**Figure 4:**
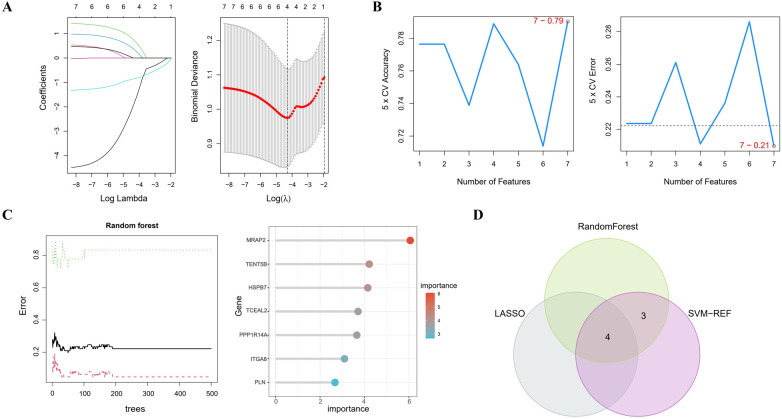
Identifying key genes using machine learning techniques. **(A)** LASSO regression analysis. **(B)** Feature selection using the SVM-RFE method. **(C)** RF algorithm application. **(D)** Venn diagram representing the shared genes across the 3 approaches.

In Figure 4A, the LASSO regression method identifies 4 genes initially extracted from the differentially expressed AAA genes. Next, the SVM-RFE algorithm identified a set of 7 genes (Figure 4B). Following this, the RF algorithm identified seven genes with an importance score exceeding 2 (Figure 4C). The results from these 3 methods were then combined using a Venn diagram, ultimately yielding 4 overlapping genes, specifically MRAP2, PPP1R14A, PLN and TENT5B (Figure 4D).

### 3.5 Validation of feature genes

**Figure 5:**
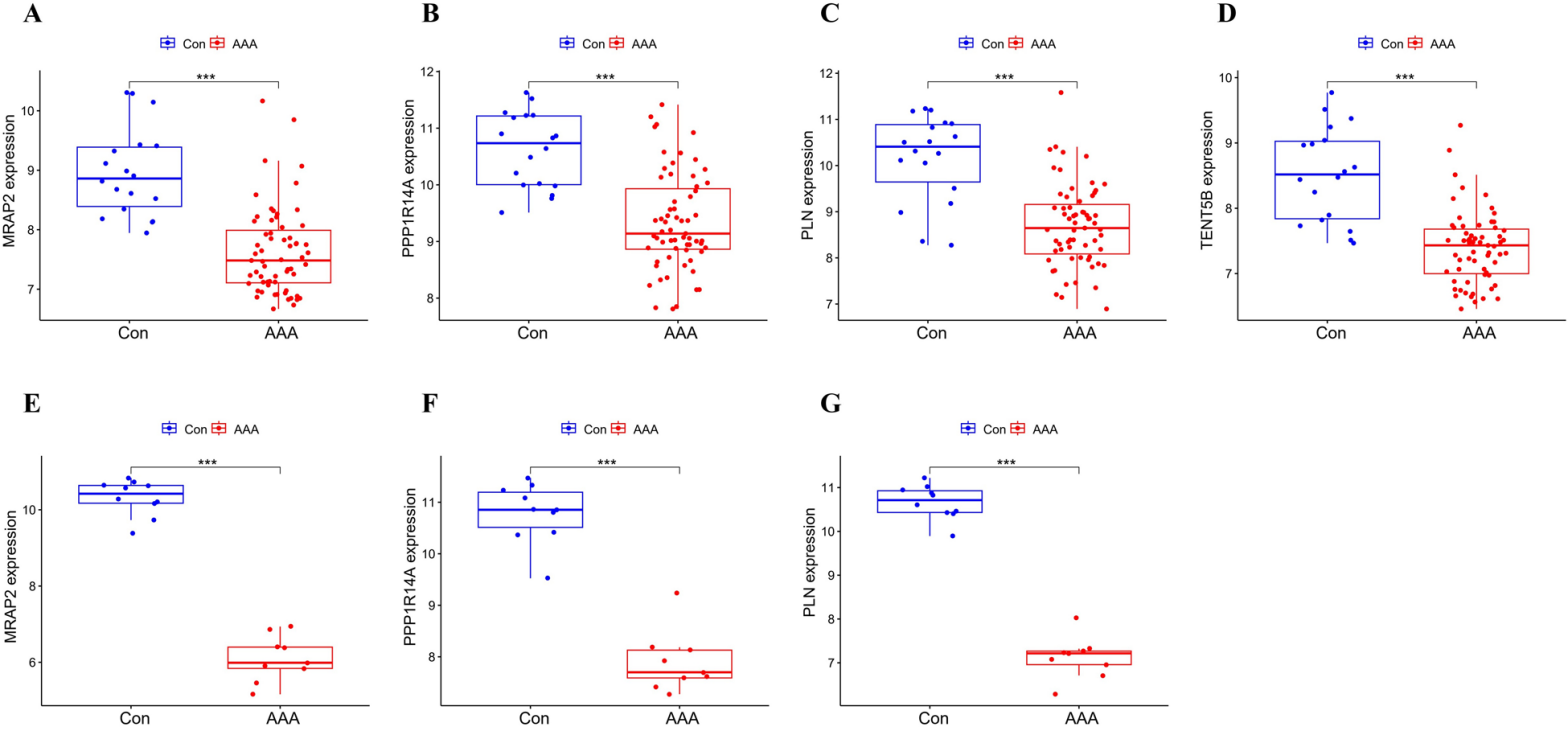
Box plot depicting the differential expression of key genes between AAA and control groups. **(A–D)** Feature genes’ expression within the training dataset. **(E–G)** Feature gene expression in the validation dataset. Significance levels: *p < 0.05, **p < 0.01, and ***p < 0.001.

MRAP2, PPP1R14A, PLN and TENT5B were found to be significantly less expressed in AAA patients than in the control group in the training dataset (*p* < 0.001) (Figures 5A–D). Next, we validated these genes using the GSE7084 dataset, which also showed reduced expression in AAA patients. A marked decline in MRAP2 expression was identified in AAA samples compared to controls (*p* < 0.001) (Figure 5E). In the same way, PPP1R14A expression was notably diminished in AAA samples (*p* < 0.001) (Figure 5F). PLN expression also showed a notable decrease in AAA samples relative to the comparator group (*p* < 0.001) (Figure 5G). However, the gene TENT5B was missing in the validation set (GSE7084), likely due to temporal and technological differences. Moreover, MRAP2, PPP1R14A, and PLN were also singled out as feature genes for more in-depth study.

### 3.6 Diagnostic efficacy of feature genes

**Figure 6:**
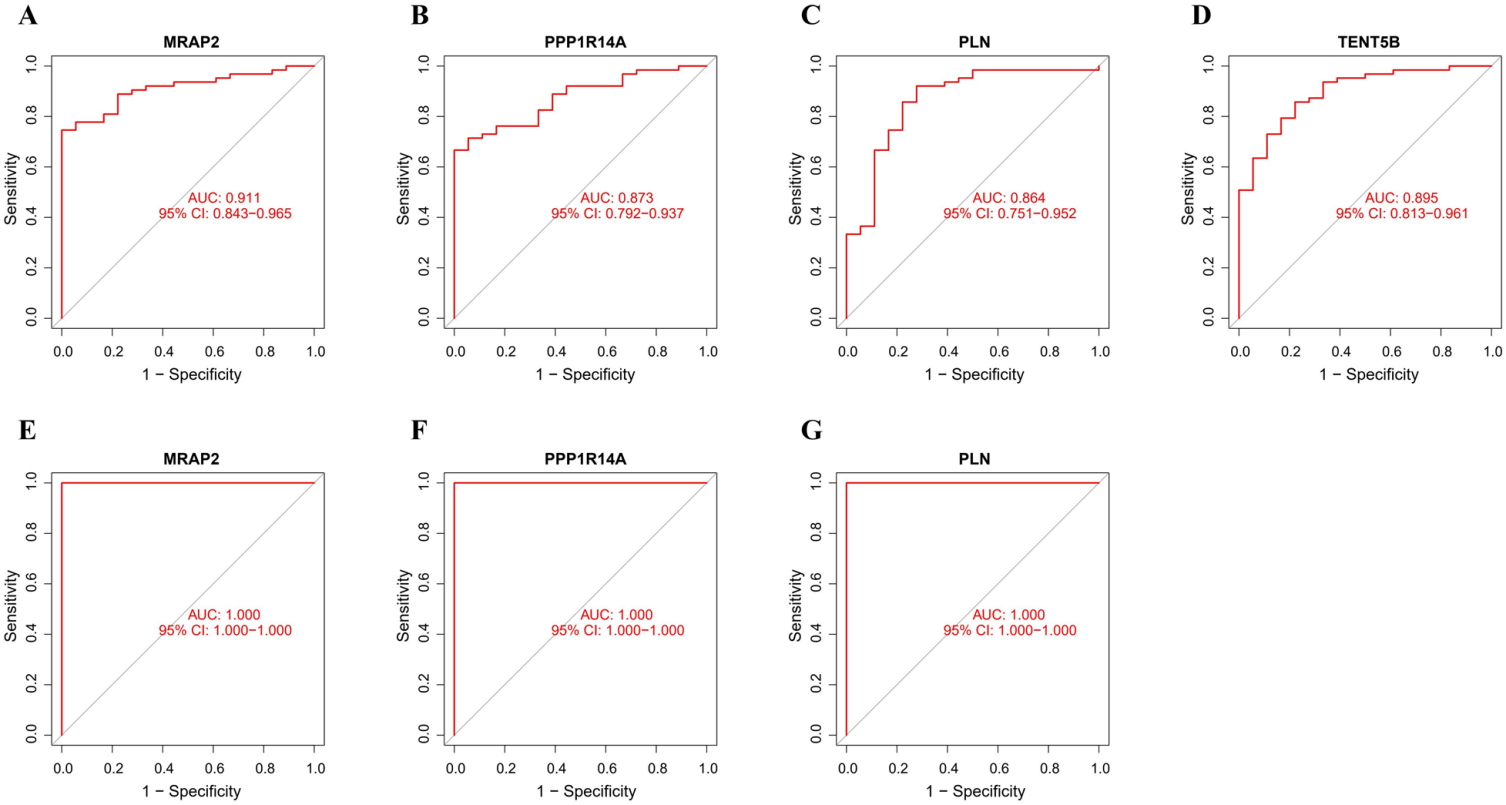
ROC curve analysis of feature genes. **(A–D)** The feature genes in the training dataset underwent ROC curve analysis. **(E–G)** The feature genes in the validation dataset underwent ROC curve evaluation.

The ROC analysis in the training group demonstrated that MRAP2, PPP1R14A, PLN, and TENT5B could efficiently discriminate AAA from controls, with AUCs of 0.911 (95% CI: 0.843–0.965) for MRAP2 (Figure 6A), 0.873 (95% CI: 0.792–0.937) for PPP1R14A (Figure 6B), 0.864 (95% CI: 0.751–0.952) for PLN (Figure 6C), and 0.895 (95% CI: 0.813–0.961) for TENT5B (Figure 6D). However, the gene TENT5B was missing in the validation set (GSE7084). In the validation group, the ROC curves demonstrated that these genes, MRAP2, PPP1R14A, and PLN, had a high predictive capacity for AAA, as shown by AUC values above 85% (Figures 6E–G), indicating strong diagnostic ability.

### 3.7 Immune infiltration analysis

**Figure 7:**
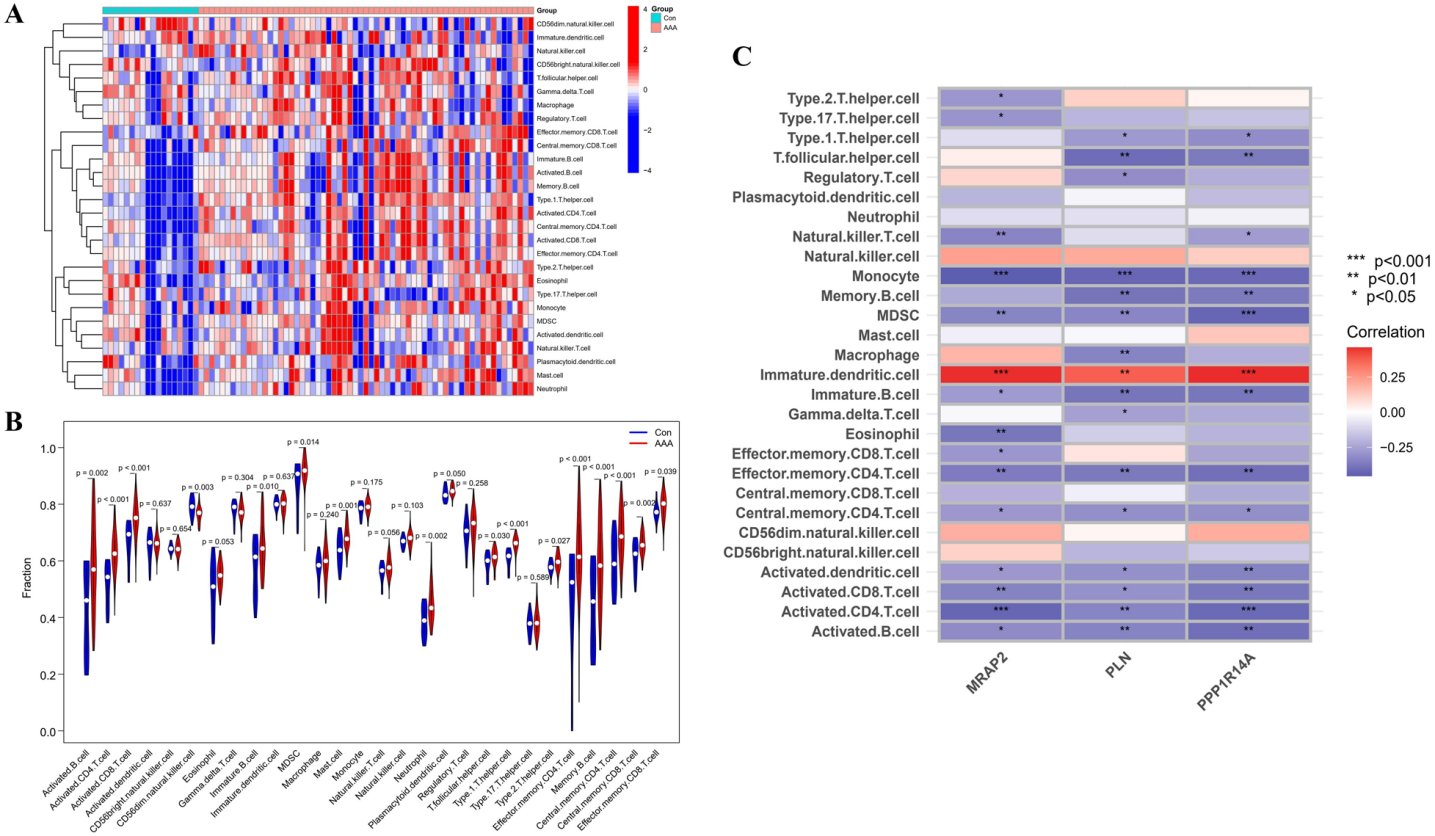
ssGSEA immune infiltration related to AAA. **(A)** Heatmap visualizing the differences in the distribution patterns of 28 immune cell populations per sample. **(B)** Variations in immune cell infiltration levels between AAA and normal control tissues. **(C)** The relationship between immune cell infiltration and MRAP2, PLN, and PPP1R14A is analyzed, with significance thresholds indicated as *p < 0.05, **p < 0.01, and ***p < 0.001.

Immune infiltration disparities between AAA patients and healthy controls were further analyzed in the study via ssGSEA analysis. Figure 7A revealed how 28 immune cells were distributed within the training group. The infiltration levels of type 2 T helper cell (Th2), type 1 T helper cell (Th1), T follicular helper cell (Tfh), neutrophil, memory B cell, Myeloid-Derived Suppressor Cell (MDSC), mast cell, immature B cell, effector memory CD8 T cell, effector memory CD4 T cell, central memory CD8 T cell, central memory CD4 T cell, activated CD8 T cell, activated CD4 T cell and activated B cell were notably higher in AAA samples. On the other hand, CD56dim natural killer (NK) cell infiltration in AAA samples showed a marked reduction (Figure 7B). In our research, we analyzed the correlation between the genes MRAP2, PLN, and PPP1R14A and various types of immune cells (Figure 7C). MRAP2, PLN, and PPP1R14A were positive associated with immature dendritic cell (DC) and were negatively correlated with monocyte, MDSC, immature B cell, effector memory CD4 T cell, central memory CD4 T cell, activated DC, activated CD8 T cell, activated CD4 T cell and activated B cell.

## 4 Discussion

AAAs pose a significant threat to health and well-being. The pathophysiological process of AAA involves a series of complex molecular and cellular events, including changes in the biomechanics of the vessel wall, thrombosis, apoptosis, extracellular matrix degradation, inflammatory responses, and vascular aging. These factors interact with each other, collectively contributing to the onset and progression of AAA. Despite a continuous stream of research on the subject, the exact causes of AAA remain not fully understood.

Although the advancement in surgical treatments, the risk of complications and mortality remains high. Understanding the underlying causes and progression of AAA is essential for developing more effective diagnostic and therapeutic approaches. While surgery is a crucial component of treatment, there is a pressing need for targeted interventions to prevent AAA development and improve outcomes. In this study, we integrated differential analysis with WGCNA to identify key genes, followed by the application of LASSO, SVM-RFE, and RF to filter potential genes. We then conducted functional and immune analyses on the selected targets. These biomarkers have the potential to enhance disease diagnosis, guide therapy selection, and predict treatment response.

In the present study, the GSE47472 and GSE57691 datasets were downloaded from the GEO database and integrated to generate a training dataset, which included 63 samples from AAA patients and 18 from healthy controls. We identified 72 DEGs in total, with an equal distribution of upregulated and downregulated genes. As per the GO and KEGG enrichment analysis outcomes, DEGs are mainly implicated in mononuclear cell differentiation, T cell activation, and IL-17 and TNF signaling pathways. These results align with prior studies (28), which have well-documented the critical roles of immune system dysregulation and inflammation in AAA progression. The diseases enriched by the DEGs, as shown by DO enrichment, were largely associated with pre-eclampsia, primary immunodeficiency disease, head and neck carcinoma, cervical cancer, aortic aneurysm and so on. Although some diseases are not directly related to AAA, the analysis highlights shared pathological mechanisms, particularly those related to immune system dysregulation and chronic inflammation (29, 30).

WGCNA has been successfully applied in earlier research to explore the links between genomic modules and clinical attributes, leading to the discovery of key genes associated with specific trait (31). We performed WGCNA to discover gene modules with correlated expression related to AAA. Subsequently, 4 feature genes were identified by finding the overlap between the genes discovered through WGCNA and the DEGs.

The deep integration of machine learning and bioinformatics has unlocked new opportunities for identifying key feature genes and predicting disease states. LASSO makes the model sparse by selecting the most important genes, which helps prevent overfitting and enhances both interpretability and generalizability (32). SVM-RFE recursively optimizes feature selection, performing well on small sample datasets and capturing complex nonlinear relationships between genes (33). RF is advantageous due to its high feature importance assessment capability, allowing it to handle nonlinear relationships in gene expression data while effectively reducing the impact of noise (34). Leveraging the complementary strengths of these methods, I combined LASSO, SVM-RFE, and RF to discover characteristic genes linked to AAA.

Then, we applied 3 different machine learning methods to filter the co-expressed genes, identifying 4 candidate genes (MRAP2, PPP1R14A, PLN, TENT5B) for AAA. The GSE7084 dataset was used to confirm the expression levels of the four genes. Several factors may explain the absence of the TENT5B gene in the GSE7084 dataset. It is likely due to the fact that the validation and test datasets were generated on different platforms and the validation dataset being collected earlier than the test dataset (35). Moreover, TENT5B, also known as FAM46, was discovered after the compilation of the GSE7084 dataset (36). This chronological discrepancy provides a reasonable explanation for its absence from the dataset. The remaining three genes (MRAP2, PPP1R14A, PLN) exhibited a significant reduction in expression in AAA tissues, which was consistently observed in the training dataset. Additionally, by performing ROC curve analysis on MRAP2, PPP1R14A, and PLN, we determined that each of them have outstanding diagnostic performance.

MRAP2 regulates energy balance and appetite through melanocortin receptors, particularly MC4R, affecting food intake and body weight. Mutations in MRAP2 are linked to obesity and metabolic disorders (37). PPP1R14A produces a protein that inhibits protein phosphatase 1, a key enzyme in muscle contraction and cell division, especially important in smooth muscle function (38). PLN regulates calcium uptake in cardiac muscle cells by inhibiting SERCA, and its phosphorylation allows proper heart muscle relaxation (39). Current research has not clearly demonstrated a direct association between the MRAP2, PPP1R14A, and PLN genes with AAA. Obesity and metabolic disorders may be risk factors for AAA, with MRAP2 regulating appetite and energy balance, potentially indirectly affecting AAA-related risk factors such as hypertension and atherosclerosis (40, 41). PPP1R14A regulates smooth muscle contraction, which is fundamental to the structure of arterial walls. Dysfunction in smooth muscle may impact the structural integrity and elasticity of arterial walls, potentially contributing to AAA (4). PLN is primarily associated with heart disease, but abnormalities in calcium regulation could affect smooth muscle function in arterial walls, thereby indirectly influencing the risk of AAA (42). Future research will likely focus more on these potential connections.

The progression of AAA is marked by an overactivation and impairment of immune cells, which contribute to the worsening of the disease. Gaining deeper insight into the mechanisms controlling immune cell activation in AAA will offer valuable targets for treatment strategies. Previous studies (43) have only analyzed the recruitment of 22 immune cell types within AAA, whereas our ssGSEA analysis provided a more comprehensive evaluation of the recruitment of 28 immune cell varieties, revealing the complexity of the immune microenvironment in AAA. The results show that AAA samples had distinctly higher levels of 15 immune cell types, such as Th2, Tfh, MDSC, mast cells, among others.

The irregular activation of immune cells, including CD8 T cells, CD4 T cells, and B cells, is a hallmark of AAA pathology. By secreting inflammatory molecules and regulating immune reactions, these cells speed up the degradation of the aortic wall (44). Tfh, a key subgroup of CD4 T cells, are fundamental in triggering germinal center formation and aiding B cell survival, differentiation, and growth. Studies show that Tfh cells may directly impact AAA through mechanisms related to inflammation (13). Similarly, Th2 cells contribute by producing IL-4 and IL-5, which induce vascular smooth muscle cell apoptosis and weaken the aortic wall. They also promote eosinophil recruitment and drive the degradation of elastin and collagen, accelerating aneurysm progression (45). Mast cells are initiators of the inflammatory response in AAA, and their activation drives the progression of the disease (46). Their activation releases proteolytic enzymes such as tryptase and chymase, which degrade elastin and collagen, contributing to tissue remodeling and further weakening the aortic wall (47). MDSCs facilitate the development of AAA through the IL-3-ICOSL-ICOS signaling axis (48). Our results also show the significant reduction in CD56dim NK cells indicates a diminished innate immune surveillance capacity, which may impair the body's ability to regulate abnormal cellular activities within the aneurysm. Immune cells gather in significant numbers at the AAA site, suggesting that the body has triggered a complex immune response that accelerates the disease's progression (49, 50). Our findings largely align with previous research. However, in contrast to earlier studies, we did not find marked variations in the levels of DCs, NK cells, and Th17 cells between the two groups. These differences may be ascribed to variations in the datasets employed or potential data imbalances in the prior research.

The correlation of genes such as MRAP2, PLN, and PPP1R14A with various immune cells further suggests that these genes might modulate immune cell function, influencing the chronic inflammatory environment in AAA. The initiation and progression of AAA rely equally on both innate and adaptive immune responses (51). The results indicate that various immune cells are closely related to AAA, directly demonstrating the widespread activation of the immune system within aneurysmal tissue. This imbalance between heightened adaptive immunity and reduced innate regulation could be key to understanding the disease progression of AAA and identifying potential therapeutic strategies.

Despite utilizing various bioinformatics techniques to identify feature genes, several important limitations must be acknowledged. First and foremost, the challenge of acquiring abdominal aorta specimens could restrict the potential clinical applications of this diagnostic model. Secondly, with a limited sample size, the reliability of the results was somewhat compromised, pointing to the need for a larger sample. Thirdly, this research utilized data from publicly available databases, which somewhat limited our ability to obtain more clinically pertinent data. The range of patient demographics and clinical features may have affected the analysis outcomes, while environmental factors could also compromise the accuracy of the susceptibility gene-based diagnostic model. Last but not least the feature genes and related immune cells identified in this study hold potential value in the diagnosis and treatment of AAA, but further validation is required.

## 5 Conclusion

MRAP2, PLN, and PPP1R14A were identified as feature genes in AAA. These genes are linked to immune cell activity, contributing to the inflammatory microenvironment that drives AAA progression. These findings highlight potential targets for developing risk predictors and immune-based therapies for AAA.

## Data Availability

The datasets presented in this study can be found in online repositories. The names of the repository/repositories and accession number(s) can be found in the article/Supplementary Material.
