# Supplementary material for: Feature genes identification and immune infiltration assessment in abdominal aortic aneurysm using WGCNA and machine learning algorithms
Source: Front Cardiovasc Med. 2024 Nov 12;11:1497170. doi: 10.3389/fcvm.2024.1497170 (PMC11588672; doi:10.3389/fcvm.2024.1497170)
Supplement: Supplementary file 1 [file Datasheet1.docx]

## Supplementary Figures


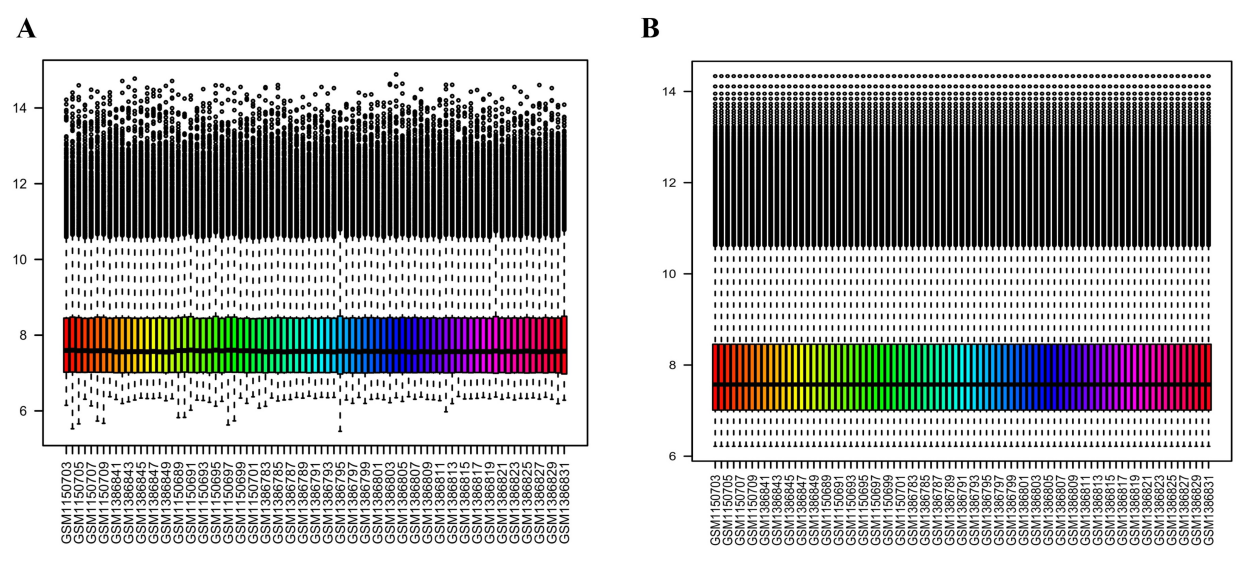


Supplementary Figure S1

Equalizing gene expression values in the dataset samples. (A) Data before normalization (B) Data after normalization.


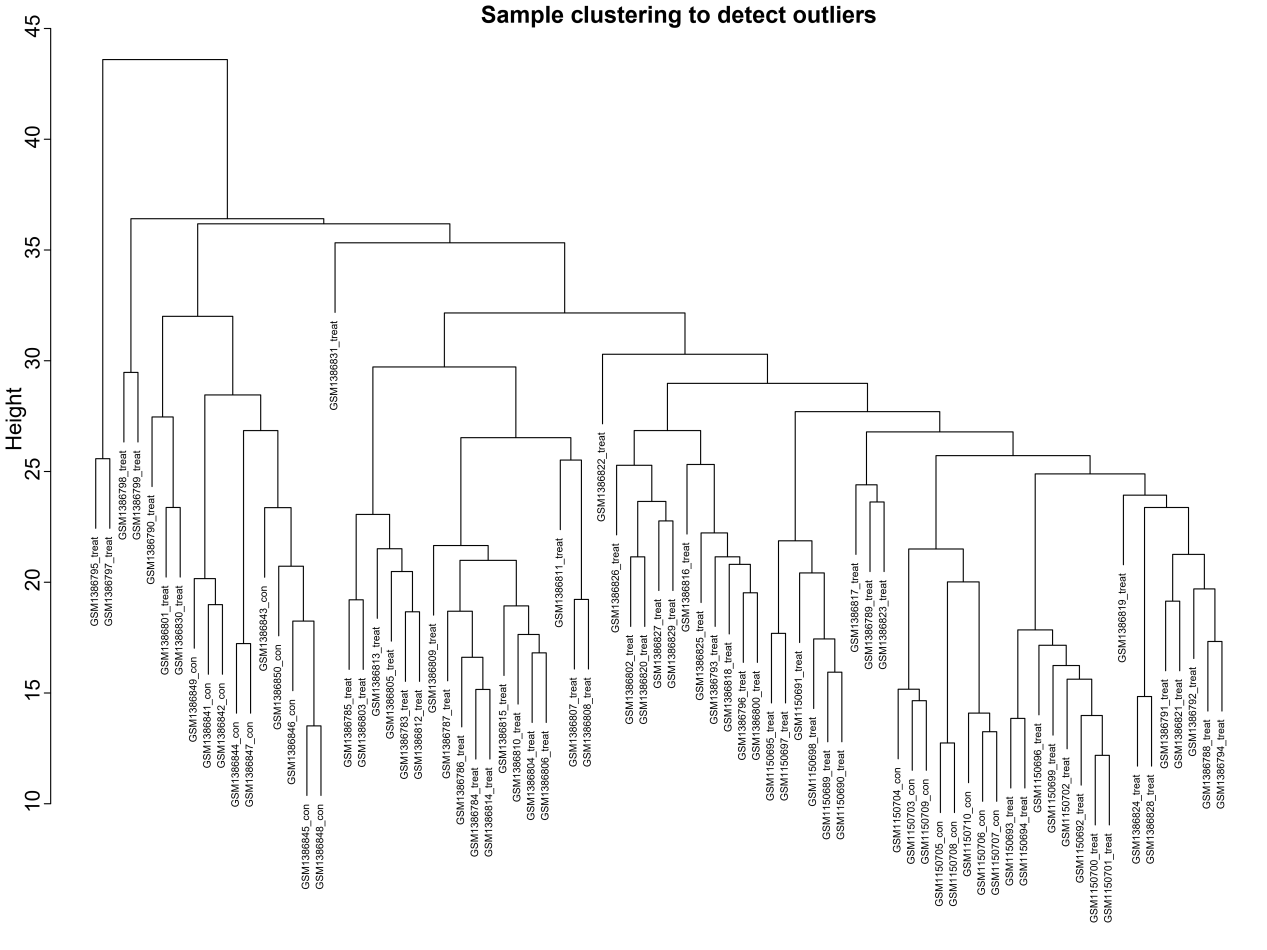


Supplementary Figure S2

WGCNA analysis applied to AAA. Two clusters were formed from the samples, and each was analyzed separately.
